# A Mixed Methods Study of Hysterectomy in a U.S. Sample of Deaf Women Who Use American Sign Language

**DOI:** 10.1089/whr.2021.0081

**Published:** 2021-11-29

**Authors:** Katja Jacobs, Ai Minakawa, Sowmya R. Rao, Poorna Kushalnagar

**Affiliations:** ^1^ Center for Deaf Health Equity, Gallaudet University, Washington, District of Columbia, USA.; ^2^ University of Massachusetts Medical School, Boston, Massachusetts, USA.

**Keywords:** deaf, sign language, women, hysterectomy, communication

## Abstract

***Background:*** Hysterectomy is one of the most common procedures performed in the United States. Yet, we know nothing about deaf women's experiences with hysterectomy. The study aims to establish a prevalence of hysterectomy among deaf women and provide insight into the experiences of those who have undergone hysterectomy.

***Materials and Methods:*** Quantitative data (*n* = 195; 27% Black, Indigenous, People of Color) were collected through a bilingual online patient-reported outcomes survey and reproductive health questions from the National Health and Nutrition Examination Survey (NHANES) between November 2019 and March 2020. Semistructured interviews were conducted between March and April 2021 with a smaller sample of deaf women who underwent hysterectomy. A multivariable logistic regression model identified the relationship between health care history and sociodemographic factors, while qualitative interview data were used to understand deaf women's experiences with hysterectomy.

***Results:*** Of the 195 deaf respondents, 34% underwent hysterectomy (*n* = 67). Results indicated that the odds of hysterectomy increased for higher age (per year), being African American/Black or Latinx, being married or living with a partner, being overweight or obese, and if communicating with the doctor through English writing or others. Qualitative interviews were conducted with eight women who provided consent to participate. Although all women reported improved quality of life posthysterectomy, patient-centered experience and decision making before hysterectomy were highly dependent on access to communication, information sources, and social support.

***Conclusions:*** Prioritizing the needs of deaf women leading up to, during, and after hysterectomy has the potential to improve overall experience with hysterectomy and patient–clinician communication.

## Background

Hysterectomy is the second most common major surgical procedure performed among women in the United States, with an estimated 600,000 hysterectomies performed annually.^1^ English-based national surveys have long excluded deaf and hard-of-hearing people who primarily use American Sign Language (ASL) (henceforth referred to as “deaf”),^2^ explaining the absence of research on the prevalence of hysterectomy among deaf populations.

Several studies reported that hysterectomy is associated with substantial improvement in quality of life.^3–5^ Among those who choose to undergo surgery with the resulting removal of ovaries, menopausal hormone therapy (MHT) is a treatment option to maintain typical estrogen levels until natural menopause. The number of deaf women who use ASL and receive MHT is unknown.

Of 45 deaf women in a 2002 focus group (69% without a college degree), a majority demonstrated a lack of understanding, or a negative perception, of women's health services, including MHT.^6^ Researchers speculated that this stemmed from communication barriers, including a lack of interpreters and clinicians' lack of experience working with deaf patients. Deaf sign language users face more difficulty with accessing health information compared with people who can hear and whose primary language is English.^7^

One possible explanation for the disparity is deaf people's limited opportunities to encounter health-related incidental learning in speaking public spaces such as radios, podcasts, and commercials without captions.^8^ Clinicians who do not effectively break down communication barriers, which can be achieved by providing accessible forms of communication, limit the opportunity for deaf women to understand how to best care for their health. For instance, the 2002 study found that, as a consequence of poor patient-centered care and associated communication, deaf women are prevented from making fully informed decisions about their own health.^6^

Another study also reported feelings of uncertainty among deaf women who were diagnosed with breast cancer due to the doctor's lack of understanding about deaf people's needs.^9^ Ensuring patients engage in health-related discussion and collaborative decision making with their doctors could improve long-term health outcomes. When patients are more engaged in shared decision making, they are more likely to feel confident in their ultimate decision.^10,11^

In an epidemiological study of NHIS administered to a total of 42,842 women (43% had disabilities), women with multiple disabilities were more likely to have a hysterectomy than women with one type of disability or no disability; this risk is heightened for women with disabilities who were under age 46.^12^ This study grouped all women, including those who were deaf, in a single disability group, thus masking likelihood ratios specific to deaf women who use ASL. Hysterectomy-related studies on women with physical and cognitive disabilities, including deafness, are scarce.

Women who have cognitive disabilities in the United States were found to be more likely to have experienced sterilization and hysterectomy at younger ages (15–44) than women with noncognitive disabilities and women without disabilities.^12^ When viewed through the lens of the U.S. eugenics movement, including the nonconsensual sterilization of over 60,000 American women between 1907 and 1963, the disparities are seen as systematically engrained in health care today.^13^ It would be remiss not to reflect on the history of sterilization among people with disabilities and emphasize the need for autonomy in health-related decision making, especially for deaf women.^14^

There is a lack of patient-reported data on cisgendered deaf women and their experiences with hysterectomies. We address this gap through a fully accessible bilingual ASL and English national survey of deaf women in the United States. The purpose of this study is to establish a prevalence of hysterectomy among deaf women in the United States, evaluate characteristics that might be associated with hysterectomy, and to provide insight into experiences of those who have undergone hysterectomy. The hysterectomy data are supplemented by qualitative interview data gathered from a small number of deaf women who consented to interviews.

## Methods

### Study design

This study uses explanatory sequential design, with quantitative data drawn from Patient Reported Outcomes Measurement Information System-Deaf Profile and reproductive health questions from National Health and Nutrition Examination Survey (NHANES), supplemented by qualitative data from semistructured interviews with a smaller sample of deaf women who have undergone hysterectomy.^15^ Following the institution's human subjects' Review Board's approval, deaf women were recruited through purposive sampling between November 2019 and March 2020.

After they filled out an appointment form, a project coordinator contacted them to schedule an interview consisting of two parts: (1) filling out demographics through an online survey that is fully accessible in ASL and English, and (2) completing a face-to-face interview with a deaf female research staff fluent in ASL. The face-to-face interview could be completed either in person or through a videoconferencing platform. The total time spent on completing the demographics and NHANES interview was 1 hour or less, and each person was given a $25 gift card for their participation.

A smaller subset of deaf women who had undergone hysterectomy were invited back to participate in a semistructured interview with a bilingual, female interviewer between February 2021 and March 2021. Interviewees gave consent to proceed with an hour-and-a-half-long interview in ASL, conducted through Zoom or videophone. Zoom interviews were recorded for later transcription by the interviewer. For videophone interviews that were not recorded, detailed notes were taken concurrently during interviews. Participants received a $50 gift card for participating.

### Quantitative analysis plan

Sociodemographic variables include age, education, race/ethnicity, marital status, sexual orientation, income, and language preference. Health indicators include health status, having a regular clinician, having insurance, and chronic medical history. Other characteristics include BMI and communication with a doctor.

Analysis included only complete data and was conducted in SAS 9.4 (SAS Institute, Inc., Cary, NC). Summary statistics (proportions, means, and standard deviations) of sociodemographic and health sample characteristics were obtained for the full female sample, with or without hysterectomy. Bivariate analysis was conducted to test the association of characteristics and PROMIS physical health/mental health/communication health *t*-scores with having a hysterectomy using a Fisher's exact test (categorical variables) or *t*-test (continuous variables).

Odds ratios (ORs) and 95% confidence intervals (CIs) were obtained from multivariable logistic regression model to evaluate the association of having hysterectomy with age (continuous), race/ethnicity (White [Ref], African American/Black, Asian/other, Hispanic), education (high school degree [Ref], some college, college graduate), marital status (divorced/widowed/separated/never married [Ref], married/living with partner), BMI (underweight/normal [Ref], overweight, obese), and communication with doctor (through a professional interpreter/directly in sign language [Ref], talking or writing in English/other).

Further analysis was limited to data from women who self-reported having had a hysterectomy (partial or full) with either or both ovaries and uterus removed. A two-sided *p*-value <0.05 was considered as statistically significant.

### Qualitative analysis plan

A bilingual researcher fluent in both ASL and English transcribed Zoom recordings into written English. All transcriptions were checked for accuracy by a study investigator who is bilingual in ASL and English. Once the study investigator approved each transcription, the Zoom recordings were deleted.

Two interviewers coded each transcript for the following themes: communication experiences with their Obstetrician/Gynecologist (OB/GYN), decision making, information about hysterectomy, and perceived quality of life before and after hysterectomy. All codes were cross-referenced to identify the most common themes. Some narratives were quantitatively coded and others were used qualitatively to support study findings.

## Results

### Sociodemographic characteristics of quantitative survey sample

As shown in Table 1, 195 deaf women (61% White; 66% heterosexual/straight; 42% with a college degree) answered questions about hysterectomy. About 19% reported having an additional disability other than being deaf or hard of hearing. Although a majority reported having health insurance of any kind (95%), only 53% reported having a provider that they saw regularly. When asked about cancer, 18% confirmed a lifetime diagnosis. About 78% reported using interpreters to communicate with their doctors and 22% communicated using spoken language, texting, paper-pen writing, or gesturing with their doctors.

**Table 1.** Distribution of Characteristics of 195 Women—Overall and by Hysterectomy Status

Variable	Overall	Hysterectomy status		
		Yes (*n* = 67)	No (*n* = 128)	
	*n*^b^ (Col%)	*n*^b^ (Row%)		*p* ^a^
Age in years				<0.0001
35–49	27 (13.1)	4 (16.0)	21 (84.0)	
50–64	90 (43.7)	23 (27.4)	61 (72.6)	
65–74	55 (26.7)	20 (37.7)	33 (62.3)	
75+	34 (16.5)	20 (60.6)	13 (39.4)	
Race/Ethnicity				0.03
White	125 (60.7)	49 (40.8)	71 (59.2)	
African American/Black	21 (10.2)	6 (31.6)	13 (68.4)	
Asian/other	32 (15.5)	4 (13.3)	26 (86.7)	
Latinx	28 (13.6)	8 (30.8)	18 (69.2)	
Education				0.28
HS degree	79 (38.5)	29 (39.7)	44 (60.3)	
Some college	39 (19.0)	15 (38.5)	24 (61.5)	
College graduate	87 (42.4)	23 (28.0)	59 (72.0)	
Sexual orientation				0.87
Straight	129 (66.2)	41 (33.9)	80 (66.1)	
LGBQA	66 (33.8)	23 (35.9)	41 (64.1)	
Preferred language				0.64
Signed language	121 (59.3)	38 (33.0)	77 (67.0)	
Both ASL and English	83 (40.7)	29 (37.2)	49 (62.8)	
Marital status				0.03
Married/living with partner	96 (46.6)	39 (42.4)	53 (57.6)	
Divorced/widowed/separated/never married	110 (53.4)	28 (27.2)	75 (72.8)	
Income range				0.29
$0–$19,999	51 (26.6)	18 (37.5)	30 (62.5)	
$20,000–$49,999	64 (33.3)	27 (43.5)	35 (56.5)	
$50,000–$99,999	46 (24.0)	14 (33.3)	28 (66.7)	
$100,000 or more	31 (16.1)	7 (23.3)	23 (76.7)	
BMI				<0.0001
Underweight/normal weight	62 (31.5)	13 (23.2)	43 (76.8)	
Overweight	60 (30.5)	31 (52.5)	28 (47.5)	
Obese	75 (38.1)	22 (31.0)	49 (69.0)	
Have health insurance				1.00
No	11 (5.4)	3 (33.3)	6 (66.7)	
Yes	191 (94.6)	62 (34.1)	120 (65.9)	
Have regular provider				0.80
No	37 (47.4)	10 (30.3)	23 (69.7)	
Yes	41 (52.6)	14 (35.9)	25 (64.1)	
Communicate with doctor				0.26
Through a professional interpreter/directly in sign language	145 (77.5)	43 (31.9)	92 (68.1)	
Talking/writing in English/other	42 (22.5)	17 (41.5)	24 (58.5)	
Additional disability other than deaf/hard of hearing				0.84
No	157 (81.3)	53 (35.1)	98 (64.9)	
Yes	36 (18.7)	10 (32.3)	21 (67.7)	
Ever had cancer				0.21
No	166 (82.2)	52 (32.3)	109 (67.7)	
Yes	36 (17.8)	14 (45.2)	17 (54.8)	
Functional hearing				0.38
Understand all/most	48 (23.4)	18 (40.0)	27 (60.0)	
Understand some/little/none	157 (76.6)	49 (32.9)	100 (67.1)	
Full hysterectomy	36			
White (average hysterectomy age = 48)		25 (69.4)	-	
Black (average hysterectomy age = 36)		4 (11.1)	-	
Asian (average hysterectomy age = 56)		2 (5.6)	-	
Latinx (average hysterectomy age = 42)		5 (13.9)	-	
Partial hysterectomy	31			
White (average hysterectomy age = 44)		24 (80.0)		
Black (average hysterectomy age = 49)		2 (3.3)		
Asian (average hysterectomy age = 36)		2 (6.7)		
Latinx (average hysterectomy age = 43)		3 (10.0)		

	n; mean (standard deviation)			
PROMIS physical *t*-score	169; 47.2 (6.1)	56; 47.5 (6.0)	103; 47.1 (6.3)	0.69
PROMIS mental *t*-score	170; 46.1 (5.0)	56; 45.8 (4.7)	104; 46.4 (5.2)	0.48
PROMIS communication health *t*-score	157; 54.9 (8.9)	52; 56.5 (9.2)	96; 54.2 (9.0)	0.14

^a^ Based on Fisher's exact test or *t*-test.

^b^ Might not add up to the total due to missing data.

ASL, American Sign Language; BMI, body mass index; HS, high school; LGBQA, Lesbian, Gay, Bisexual, Queer, Asexual; PROMIS, Patient Reported Outcomes Measurement Information System.

With exception for BMI (higher for hysterectomy group), marital status (higher for married/living with partner), and age (older for hysterectomy group), no meaningful group differences were observed across hysterectomy status for any of the demographic and health variables. Within the hysterectomy group (*n* = 67), about 54% had both ovaries removed. The African American/Black women group had the youngest average age of 36 years for full hysterectomy, whereas Asian women had the youngest average age of 36 years for partial hysterectomy. Distribution of physical, mental, and communication health outcome scores were similar for the hysterectomy and nonhysterectomy groups.

Results from multivariable logistic regression model indicates that the odds of hysterectomy increased for higher age (per year) (OR [95% CI]: 1.035 [0.998–1.074]; *p*-value = 0.06), being African American/Black (1.243 [0.376–4.111]) or Latinx (1.253 [0.399–3.929]), being married or living with a partner (2.941 [1.392–6.214]), being overweight (3.165 [1.251–8.008]), or obese (1.096 [0.431–2.786]) (overall *p*-value = 0.01), and if communicating with the doctor through English writing or others (1.250 [0.521,3.001]; *p*-value = 0.62. The odds decreased for Asian/other (0.273 [0.078–0.954]) and those who had some college (0.937 [0.349–2.519]) or were college graduates (0.531 [0.230–1.224]).

The *p*-values for Race/Ethnicity and education were 0.17 and 0.28, respectively (Table 2). The overall model had good fit with a c-statistic of 0.748.

**Table 2.** Odds Ratios and 95% Confidence Intervals from Multivariable Logistic Regression Model to Predict Having Hysterectomy

Characteristic	OR (95% CI)	*p* ^a^
Age in years	1.035 (0.998–1.074)	0.06
Race/Ethnicity		0.17
White	Ref.	
African American/Black	1.24 3 (0.376–4.111)	
Asian/other	0.273 (0.078–0.954)	
Latinx	1.253 (0.399–3.929)	
Education		0.28
HS degree	Ref.	
Some college	0.937 (0.349–2.519)	
College graduate	0.531 (0.230–1.224)	
Marital status		<0.01
Divorced/widowed/separated/never married	Ref.	
Married/living with partner	2.941 (1.392–6.214)	
BMI		0.01
Underweight/normal weight	Ref.	
Overweight	3.165 (1.251–8.008)	
Obese	1.096 (0.431–2.786)	
Communication with doctor		0.62
Through a professional interpreter/directly in sign language	Ref.	
Talking or writing in English/other	1.250 (0.521–3.001)	
C-statistic = 0.748		

^a^ Based on Wald chi-square test from multivariable logistic regression model that included all variables in the table.

CI, confidence interval; OR, odds ratio.

### Sociodemographic characteristics of qualitative interview sample

As shown in Table 3, the years in which hysterectomy was performed for the eight respondents ranged from 1981 to 2019. Of the eight who had a hysterectomy (88% White; 63% with a college degree), six participants (75%) used MHT.

**Table 3.** Qualitative Interview Sample of Deaf Women Who Had Hysterectomy (*n* = 8)

Variable		
Age of hysterectomy in years (range)	37–56 years old	
Year of hysterectomy^a^	1981–2019	

	*n*	%
Race/Ethnicity		
White	7	87.5
Person of color	1	12.5
Education		
High school	0	0.0
Some college	3	37.5
College	5	62.5
MHT		
Yes	6	75.0
No	2	25.0
Tried other options before hysterectomy		
Yes	4	50.0
No	4	50.0
Quality of OB/GYN relationship		
Good to excellent	7	87.5
Poor to fair	1	12.5
Communication with OB/GYN in the doctor's office		
Through interpreter	4	50.0
Writing/texting/other	4	50.0
Communication with OB/GYN in the hospital		
Through interpreter^b^	2	25.0
Writing/texting/other	6	75.0
Communication experience with OB/GYN		
Good to excellent	5	62.5
Poor to fair	3	37.5
Decision making about hysterectomy		
Shared with doctor	6	75.0
Only doctor	2	25.0
Information receiving about hysterectomy		
Fully satisfied	3	37.5
Somewhat satisfied	3	37.5
Little or not at all satisfied	2	25.0
Quality of life before hysterectomy		
Good to excellent	0	0.0
Poor to fair	8	100.0
Quality of life after hysterectomy		
Good to excellent	8	100.0
Poor to fair	0	0.0

^a^ Four respondents underwent hysterectomy before when the ADA of 1990 was passed and five respondents underwent hysterectomy after 1990.

^b^ All respondents who used an interpreter to communicate with their OB/GYN underwent their procedure after the ADA was passed.

ADA, Americans with Disabilities Act; MHT, menopausal hormone therapy; OB/GYN, Obstetrics and Gynecology.

Five participants perceived their quality of communication with their OB/GYN as “Good to Excellent” and the remaining three participants reported overall communication experiences with OB/GYN as “Poor to Fair.” Four participants (50%) primarily used live sign language interpreters or video remote interpreting to communicate with their OB/GYN, all of whom underwent hysterectomy after 1990, when the Americans with Disabilities Act (ADA) was passed. Four participants (50%) who underwent hysterectomy before 1990 communicated in various ways, such as writing back and forth using pen and paper, texting using a phone, gesturing, lipreading, and/or relying on friends to interpret.

Participants relied on the internet, print media (*e.g.*, books, magazines, brochures), and/or their doctor's explanations to receive information about risks and benefits of hysterectomy during their decision-making process. Of the eight participants, only three (38%) were “fully satisfied” with the quality and quantity of information that they received and two participants (25%) felt “little or not satisfied.” Three respondents (38%) felt “somewhat satisfied” with the information they received.

All eight participants reported their perceived quality of life before hysterectomy as “Poor to Fair” because of pain or excessive menstruation that interfered with everyday activities and they all perceived their quality of life following hysterectomy as “Good to Excellent.”

### Access to communication

Women who had access to their preferred or most accessible method of communication—whether that be through an interpreter, using paper and pen to write to communicate, or using a speech-to-text transcription app—identified themselves as being able to better understand hysterectomy and its process. Having adequate access to communication helps with being fully informed and being able to freely discuss their health with their doctors, allowing deaf women to feel more confident and comfortable moving forward with a procedure like hysterectomy.

“…when I showed the [speech to text] app to my OB/GYN and the anesthesiologist, they liked it. It was a pretty positive experience and my nerves went away. They gave me autonomy in that situation and the freedom to decide how I wanted to communicate.” (48 years old at the time of hysterectomy, post-ADA)

“When I went back to that first interpreter that I really liked, the doctor seemed to ask me a lot more questions than with the other two interpreters. I was thinking, ‘Why didn't you listen to me before?’ The doctor said they just wanted to make sure they understood what I was saying…Yeah, they seemed to ask me a lot more questions with the first interpreter that I liked there…” (42 years old at the time of hysterectomy, post-ADA)

Furthermore, when deaf women are provided with their preferred form of communication coupled with a clinician who is patient and competent in communicating with deaf patients, the patient's understanding of their own health improves dramatically. The physician's approach to communication and demonstrated respect for the patient is an important element in full communication access.

“They were willing to write to communicate and show me images of what was normal and what wasn't normal. We were able to figure out where we needed to go from there… Communication was very clear…Looking back on the process surrounding my hysterectomy, it was a good experience, even though I had no access to ASL interpreters. I still had a pretty decent experience trying to communicate with everyone.” (37 years old at the time of hysterectomy, pre-ADA)

“She was very good at showing me things on her laptop related to my health. I was very impressed with how much time she spent with me. Some doctors speed through the appointment very quickly but I loved her.” (45 years old at the time of hysterectomy, post-ADA)

Despite speaking positively about their communication experience during a time when interpreters were not widely available, women who underwent their hysterectomy before 1990, when the ADA was passed, say that in hindsight, they wish they had an interpreter at the time and believe it would have significantly improved their overall communication experience.

“We didn't have an interpreter, nothing. I never had an interpreter when I went to the hospital and kind of just had to deal with that. I felt alone but I had to trust them.” (37 years old at the time of hysterectomy, pre-ADA)

“Looking back on the process surrounding my hysterectomy, it was a good experience, even though I had no access to ASL interpreters. I still had a pretty decent experience trying to communicate with everyone…I think if there were an interpreter there with us at the time, I may have had more information access. I could have asked more questions. At the time, I didn't think that was something I could do. I didn't even know having an interpreter was possible at the time.” (41 years old at the time of hysterectomy, pre-ADA)

These qualitative data appear to align with the “shared decision” response to the question “Can you describe your experience related to shared decision making about your hysterectomy treatment?” in that having complete access to communication improves quality of decision-making ability. These quotations were derived from the six out of eight deaf women who perceived themselves as having had a shared decision-making experience with their OB/GYN.

### Social support

Women who mentioned having social support from family and friends during the decision-making process, which includes being listened to and having opportunity to openly discuss their thoughts, shared feelings of relief, making the process surrounding their hysterectomy a more positive and pleasant experience. The presence of family members or friends during doctor's visits, surgery, and recovery also aided in feeling more comfortable and a more efficient communication experience for respondents.

Furthermore, having opportunity to listen to deaf female friends who have already undergone hysterectomy helped participants feel confident regarding their own hysterectomy.

“My husband came to all the appointments with me. It was nice to have him there as support…I felt good about [my] decision, I never felt pressured or stressed about what to do because I had my husband and doctor as support…My mom and my sister were [also] very supportive and open to discussion. My sister told me to go ahead and have the surgery… I had positive reinforcement from my [deaf] friends and [hearing] family.” (48 years old at the time of hysterectomy, post-ADA)

“My husband was there with me throughout the whole process. We worked together and supported each other. At that time, my husband encouraged me, telling me we would make it through it. If it weren't for my husband, I may have felt a hysterectomy wasn't worth it.” (37 years old at the time of hysterectomy, pre-ADA)

“I absolutely had support from my friends who helped me make a decision.” (45 years old at the time of hysterectomy, post-ADA)

### Access to information

In addition to doctors' explanations of treatments and their risks and benefits, which are crucial in the decision-making process, some women also tried to maximize understanding about their procedure through reading materials, asking doctors to explain in depth and clarify, and reaching out to someone with a similar experience. Having access to reliable and factual resources, and thus information, through the Internet or reading material allows women to make a decision that is best for their health and wellbeing.

“I did read accounts of women's experiences with hysterectomy and their stories on the Internet. A lot of the stories that I read talked about their negative experiences with surgery. I read about many failed surgeries and it seemed that women who have had positive experiences with hysterectomy don't post about it. They're more likely to post about their negative experiences. I think that's why I didn't rely heavily on reading people's experiences on the internet. My friend who had a positive experience with their hysterectomy and I were the same age, we used to be friends, we haven't experienced menopause yet, and were very similar in a lot of ways. Because of that, I decided to go ahead with the risk of having surgery. I had a better sense of what to expect than I did before.” (48 years old at the time of hysterectomy, post-ADA)

“My first doctor always made sure that I fully understood everything and that I got full access to accurate information.” (45 years old at the time of hysterectomy, post-ADA)

“All the reading material the doctors gave me was very clear…I was able to understand it.” (41 years old at the time of hysterectomy, pre-ADA)

“At the time, I read encyclopedias, books, and went to the library to learn more. I mostly depended on what the doctor explained to me, which helped. Those were the only sources of information I had at the time.” (37 years old at the time of hysterectomy, pre-ADA).

## Discussion

The prevalence of hysterectomy in our deaf women sample (*n* = 195) was 34%, with Black and Latinx women having increased odds of having a hysterectomy compared with White and Asian women. This is comparative with the national prevalence of hysterectomy at 33% according to the Centers for Disease Control and Prevention.^14^ While the rates did not show disparity in the prevalence of hysterectomy among deaf women compared with the general women population, qualitative experiences were clearly different with deaf women encountering communication-specific difficulties that impacted their overall experiences with hysterectomy.

Qualitative interview data with a smaller subsample indicated strong themes of the importance of access to communication, full access to information, and social support for deaf women navigating hysterectomy, contributing to changes in quality of life following the procedure.

Posthysterectomy quality of life among deaf women are consistent with other studies, which showed that quality of life for women who choose to undergo hysterectomy does improve.^3–5^ However, while all interviewees felt that their quality of life improved after hysterectomy, we must take into account the experiences some of these deaf women had to go through to arrive at that outcome. Experiences include barriers to communication with doctors, limited access to information about hysterectomy, and lack of support from family and friends that interfered with their autonomy when trying to engage in decision making.

Although communication accessibility (*e.g.*, interpreters, closed captions, other technology) for deaf patients has significantly improved over the past 30 years due in part of the ADA, communication barriers, for which there are more widely available solutions today, are still evident for deaf women seeking out health care services whether they received a hysterectomy in 1981 or 2019. Barriers to communication compromise a woman's ability to make a fully informed decision about her reproductive health, emphasizing the need for increased availability of accessible forms of communication in doctor's offices and hospitals.

The passing of the ADA in 1990 meant that hospitals became responsible for providing effective communication, including interpreters.^16^ It is expected that the four participants who underwent hysterectomy after the ADA was passed had interpreters during visits with their OB/GYN. Having qualified interpreters were found to have positively impacted their overall communication experiences. The remaining four interview participants who underwent hysterectomy before 1990 relied on writing using pen and paper, gesturing, or a family member or friend who interpreted to communicate with their OB/GYN.

A similar observation between these two groups, despite the differences in communication accessibility, is the positive patient-centered care that women in both groups received from their doctors. Most women in the pre-ADA group reported that their doctors ensured their understanding of the risks and benefits associated with hysterectomy and allowed them to ask as many questions as needed to alleviate their concerns. Full access to communication does not end at simply providing a preferred form of communication.

Respondents expressed having a better understanding of their health and hysterectomy when their OB/GYN knew how to communicate with a deaf person and in a way that was unique to the individual, maximizing the deaf person's understanding. Similar experiences were also mentioned by all women in the post-ADA group that received interpreting services for their clinical visits. Clinicians in both groups had the responsibility of ensuring that deaf women were adequately informed and involved in decision making about their treatment.

Deaf sign language users have historically had limited opportunities to engage in health-related discussions with physicians, leading to a lack of understanding of health-related issues.^7^ When the communication needs of deaf patients are made a priority, this can ultimately lead to improved satisfaction with services they receive and improved long-term quality of life.^17^

Having the opportunity to listen to stories from other women who had a similar experience also positively contributed to respondents' confidence in their decision making and plays into access to information, as well as social support. Discussing options with family, a partner, and other women also affected perceptions in decision making. Some women reported that it was challenging to find someone who had similar experience in the deaf community that they belong to.

We found that when deaf women know another deaf woman or, ideally, multiple deaf women who had a similar experience with hysterectomy and a shared language, they gain a better understanding of the process and feel more comfortable moving forward with a decision. A frustrating, stressful experience leading up to and during hysterectomy can negatively impact the recovery process postsurgery, which can be alleviated by social support.^18^ When the wellbeing of deaf women is ensured all throughout a process like hysterectomy, by receiving access to communication, information, and social support, the likelihood of an overall more positive experience and quick recovery improves.

In sum, deaf women receive information in different ways. Multiple sources of information are needed to make informed decisions about hysterectomy. All resources must be made accessible in ASL with captions, easy-to-read text, and infographics for those who are not fluent in one language or the other. In addition to providing accessible resources, deaf women being given autonomy in communicating with their clinicians is critical for a positive experience in navigating their health and related procedures. Doctors that demonstrated patience, provided visuals, and took their time with explanations and responding to questions affected a better understanding of the procedure among respondents.

With regard to racial disparity for hysterectomy in the general population, Black women were found to have undergone hysterectomy at higher rates than white and Hispanic women.^19^ This is inconsistent with our results, which show no differences in the rates of hysterectomy among Black deaf women compared with White and non-Black deaf women. However, caution must be made when interpreting such findings, as our sample was relatively small (*n* = 66; 26% persons of color) compared with the cited study that had a much larger, more racially and ethnically diverse sample of women. A larger study is warranted before a claim can be made about racial/ethnic disparities within the deaf and hard-of-hearing community of women who use ASL.

Deaf women are not a homogeneous entity but are rather made up of individuals with intersecting identities, varying based on race, ethnicity, socioeconomic status, sexual orientation, and language background, impacting the best ways in which they can access care and communication. Future research should consider how race, socioeconomic status, and language background plays a role in access to reproductive health care for deaf women. To investigate such a question, an adequately powered study will be required to conduct intersectional analyses. The results from this study can be used to inform health policy work to optimize information and communication accessibility for deaf women who seek reproductive health care.

### Limitations

Our study has some limitations. Although this study utilized recruitment approaches that have been found to be effective in recruiting and retaining members of hard-to-reach communities such as in our women sample, potential self-selection bias may be present. Also, the virtual face-to-face NHANES interviews may indicate self-selection bias of those who feel comfortable using the computer, tablet, or videophone to answer questions in ASL and English. The demographic characteristics in the subsample of primarily white deaf women with at least some college education who participated in a longer qualitative interview may not adequately capture the full range of lived experiences among high school educated or Black, Indigenous, People of Color deaf women who underwent hysterectomy.

However, a strength of this study is that it is the first to provide both quantitative and qualitative evidence on ASL-using deaf women's experiences with hysterectomy. The data presented in this study were gathered from hard-to-reach individuals and offers rich information for further investigation.

## Abbreviations Used

ADA: Americans with Disabilities Act
ASL: American Sign Language
BIPOC: Black, Indigenous, People of Color
CI: confidence interval
LGBQA: Lesbian, Gay, Bisexual, Queer, Asexual
MHT: menopausal hormone therapy
NHANES: National Health and Nutrition Examination Survey
OB/GYN: Obstetrics and Gynecology
OR: odds ratio
PROMIS: Patient Reported Outcomes Measurement Information System
VRI: video remote interpreting
